# The Prevalence of Thyroid Nodules and an Analysis of Related Lifestyle Factors in Beijing Communities

**DOI:** 10.3390/ijerph13040442

**Published:** 2016-04-22

**Authors:** Hua Jiang, Yongfeng Tian, Wenhua Yan, Yue Kong, Haibin Wang, Anping Wang, Jingtao Dou, Ping Liang, Yiming Mu

**Affiliations:** 1 Department of Endocrinology, Chinese People’s Liberation Army (PLA) General Hospital, 28 Fuxing Road, Beijing 100853, China; nndwmnby@gmail.com (H.J.); tianyf1988@163.com (Y.T.); yanwenhua301@163.com (W.Y.); wanghaibin2307@126.com (H.W.); anping1128@hotmail.com (A.W.); jingtaodou@sohu.com (J.D.); 2 Department of Endocrinology, the First Affiliated Hospital, Chinese People’s Liberation Army (PLA) General Hospital, 51 Fucheng Road, Beijing 100048, China; 3 Department of Ultrasonography, Chinese People’s Liberation Army (PLA) General Hospital, 28 Fuxing Road, Beijing 100853, China; guoweiweic1225@vip.sina.com (Y.K.); liangping301@hotmail.com (P.L.)

**Keywords:** thyroid nodule, epidemiology, ultrasound, smoking, lifestyle

## Abstract

Thyroid nodules (TNs) have annual increasing trends worldwide, and large-scale investigations on the prevalence of TNs in Beijing communities have not been conducted since the introduction of salt iodization in 1995. We performed a cross-sectional study to determine the prevalence of TNs, their epidemiological characteristics, and their correlation with lifestyle factors. A total of 6324 permanent residents aged 18 years or older (mean age, 52.15 ± 11.58 years) from seven representative communities in Beijing were included in the analyses. Once informed consent was obtained, the subjects were asked to complete questionnaires, a physical examination, and thyroid ultrasound. A total of 3100 cases had TNs. The overall prevalence rate was 49.0%, and the age-standardized prevalence was 40.1%, which increased significantly as age increased (*p* < 0.001). The prevalence was significantly higher in females compared to males (*p* < 0.001), and it was significantly higher among female current smokers and former smokers compared to non-smokers (*p* = 0.007). There was no correlation between alcohol consumption and TNs, and there were no significant differences in the prevalence among different groups of taste preference. The prevalence decreased with an increased frequency of seafood intake (*p* = 0.015) and with higher literacy levels (*p* < 0.001). The Cochran–Armitage trend test showed that the prevalence significantly increased with decreased physical labor and exercise intensity (*p* < 0.001, *p* = 0.009). Logistic regression analysis showed that age (Odds ratio (OR) = 1.039 (1.034–1.044), *p* < 0.001), the female sex (OR = 1.789 (1.527–2.097)), Body mass index (BMI) (OR = 1.019 (1.005–1.034)), and current smoking habits (OR = 1.246 (1.046–1.483)) were independent risk factors for TNs. Our findings indicate that there is a high prevalence of TNs in Beijing, with a higher prevalence in females than in males. Moreover, the prevalence increases as age increases. Smoking and BMI are independent risk factors for TNs. Therefore, intervention against smoking and weight loss might help reduce the risk of TN occurrence.

## 1. Introduction

Thyroid nodules (TNs) are among the common diseases of the endocrine system, with 3%–7% prevalence by palpation [1]. The prevalence by high-resolution ultrasonography among randomly selected individuals is 19%–67% [2], with annual increasing trends worldwide. 5% to 15% of TNs is thyroid cancer [3], which has become the fastest growing cancer. The high prevalence of TNs may be partly because of the advancements in diagnostic technologies, but it still cannot be explained by traditional risk factors such as sex, age, iodine intake, and radiation exposure. A number of recent studies [4,5,6] reported that obesity and metabolic syndrome were associated with increased TN prevalence. Meanwhile, lifestyles have very important effects on obesity and metabolic syndrome. Unhealthy habits have sparked an epidemic of diseases that together constitute the leading cause of death globally, as reported by the World Health Organization (WHO). Therefore, it is imperative to investigate the effects of lifestyles on the prevalence of TNs.

In 2010, the prevalence of TNs was 18.6%, based on the epidemiological survey of ten cities in China. Chen *et al.* [7] reported the prevalence of TNs among men and women was 24.1% and 34.7% in Hangzhou, a city of eastern China in 2013. The prevalence among people aged over 40 years old in Nanjing had reached 46.6% in 2014 [8]. Beijing is an area of iodine deficiency. Large-scale investigations on the prevalence of TNs in Beijing communities have not been conducted since the introduction of salt iodization in 1995 for the improvement of iodine nutrition. We investigated the prevalence of TNs in Beijing communities and analyzed its correlation to certain lifestyle factors.

## 2. Subjects and Methods

### 2.1. Subjects

The subjects of the study were recruited from August to December of 2013. A cluster random sampling method was used in this survey. Seven representative communities were randomly selected from the suburban area of Beijing. The exclusion criteria were: an age below 18 years, a lack of mobility, communication disorders, pregnancy, severe cardiac, hepatic or kidney disease, or no experience with thyroid ultrasound. People who were or had been treated with medicines influencing thyroid function, such as amiodarone, iodine, lithium preparation, interferon, or hormones such as glucocorticoid and estrogen were also excluded. A total of 8233 individuals were initially included; however, 85% participated. Another 674 people were excluded according to the criteria. Finally, 6324 participants were included in the cross-sectional survey analysis. All subjects gave their informed consent for inclusion before they participated in the study. The study was conducted in accordance with the Declaration of Helsinki. The study was approved by the ethics committee of the Chinese People’s Liberation Army (PLA) General Hospital (Project code 2011ZX09307-001-08).

### 2.2. Measurements

Initially, potential participants signed informed consent forms, and then assented to a questionnaire survey, a physical examination, and thyroid ultrasound. A thyroid nodule is a discrete lesion within the thyroid gland that is radiologically distinct from the surrounding thyroid parenchyma [9]. Thyroid ultrasound was performed using LOGIQ e (GE Healthcare, Milwaukee, WI, USA) B ultrasonic wave, a high-frequency linear array 12L, and a 7–13-MHz probe.

Smoking habits, drinking, taste preferences, seafood intake, educational level, physical labor intensity, physical exercise intensity, and medical history were recorded in the questionnaire. As far as smoking was concerned, individuals were divided into current smokers (those who had been smoking at least one cigarette per day for over six months), former smokers, and non-smokers. For alcohol-drinking status, subjects were classified as current drinkers (those who had been taking alcohol at least once a week for over six months), former drinkers, and non-drinkers. Concerning the educational level, subjects were classified into six groups: those who had never gone to school, those who had gone to elementary school, those who had gone to middle school, those who had gone to high school/technical school/polytechnic school, those who had gone to junior college, and those who had gone to college and beyond. Taste preferences were divided into three groups of salt preference: salty, average, and less salty. Frequency of seafood intake was assessed in three groups: frequent (≥3 times/week), occasional (<3 times/week), and never. According to national physical labor intensity classification GB3869-1997, physical labor was assessed and placed into four groups of very light, light, moderate, and heavy physical labor. Based on the classification of American College of Sports Medicine, physical exercise intensity was grouped into mild (exercise heart rate <55% of maximum heart rate), moderate (exercise heart rate within 55%–69% of maximum heart rate), and high (exercise heart rate ≥70% of maximum heart rate).

### 2.3. Statistical Analysis

Data were recorded on the computer twice by two independent individuals who were responsible for logic and consistency checks. Statistical analyses were performed using SPSS version 16.0 (SPSS Inc., Chicago, IL, USA). Age-standardized prevalence was calculated by the direct method using data of the population distribution in China in 2010. Measurements were expressed as mean ± standard deviation (SD), and two groups were compared using *t*-test. Counts were represented by frequency or percentage, and the groups were compared using a chi-square test and a Cochran–Armitage trend test. Related factors were analyzed using logistic regression. Missing data were missing at random and were not included in the analysis. A *p* < 0.05 was considered statistically significant.

## 3. Results

### 3.1. General Features

A total of 6324 subjects (mean age, 52.15 ± 11.58 years; male/female, 2264/4060) participated in this study. The demographic data are shown in Table 1. Age, body mass index (BMI), percentage of smokers, and percentage of alcohol drinkers among female subjects were significantly lower compared to male subjects (*p* < 0.001).

### 3.2. Epidemiological Characteristics of Thyroid Nodules

Among the 6324 cases investigated, 3100 subjects had TNs. The overall prevalence rate was 49%; after standardization by age, prevalence became 40.1%. Seventy-three patients had undergone surgical resection for TNs, and 57 patients relapsed. The prevalence of TNs was significantly higher in females compared to males (52.5% *vs.* 42.7%, *p* < 0.001). There were 1099 cases of solitary nodules, accounting for 35.5%. Multiple nodules accounted for 59.2% in males and 67.0% in females (Table 2).

**Figure 1:**
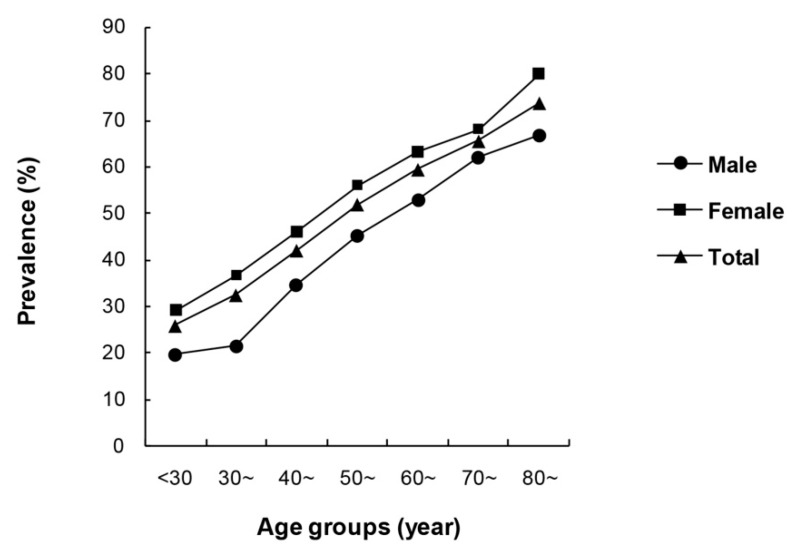
Comparison of the prevalence of thyroid nodules in different age subgroups.

The prevalence rates of TNs were 25.8%, 32.4%, 42.0%, 51.9%, 59.4%, 65.5%, and 73.7% in the less than 30, 30–39, 40–49, 50–59, 60–69, 70–79, and 80 year-old and above groups, respectively. Prevalence rates increased significantly as age increased, and the Cochran–Armitage trend test was also statistically significant (χ^2^ = 262.289, *p* < 0.001). It was the same for both males and females (*p* < 0.001) (Figure 1).

### 3.3. Correlation Analysis of Thyroid Nodules and Lifestyles

The prevalence of TNs was 43.7% in male current smokers, 42.1% in male former smokers, and 40.3% in male non-smokers, with no significant difference among the groups (χ^2^ = 1.958, *p* = 0.376). The prevalence in female current smokers and former smokers was 61.4% and 64.4%, respectively, which was significantly higher than the 51.9% in female non-smokers (χ^2^ = 9.794, *p* = 0.007). There was no significant difference in the prevalence of TNs among the current drinkers, former drinkers, and non-drinkers in either males or females (Table 3).

Individuals consuming iodized salt were 99.7%. The prevalence of TNs was 48.6%, 49.4%, and 48.8% in the salty, average, and less salty salt preference groups, respectively. The prevalence of TNs in different groups had no statistical difference (χ^2^ = 0.317, *p* = 0.854).

The prevalence of TNs was 46.1% in the group with frequent seafood intake, 48.5% in the group with occasional intake, and 52.4% (the highest) in those with no seafood intake. The differences in prevalence among these groups were statistically significant (χ^2^ = 6.087, *p* = 0.048). Moreover, with increased frequency of seafood intake, TN prevalence decreased, and the Cochran–Armitage trend test resulted in statistical significance (χ^2^ = 5.874, *p* = 0.015).

The analysis of the physical labor intensity showed that the very light manual labor group had the highest prevalence of TNs with 50.5%, followed by the light manual labor group with 45.4%, the moderate labor group with 43.1%, and finally the heavy labor group with 25.0%. The prevalence of TNs among different physical labor groups differed significantly (χ^2^ = 16.581, *p* = 0.001). Thus, with the decrease in physical labor intensity, nodular prevalence significantly increased, shown by the Cochran–Armitage trend test (χ^2^ = 15.746, *p* < 0.001).

The prevalence of TNs in the mild physical exercise intensity group was the highest with 49.6%, followed by the moderate intensity physical exercise group with 47.5%, and finally the high intensity physical exercise group with 34.4%, which was the lowest. TN prevalence significantly decreased with increased physical exercise intensity, shown by the Cochran–Armitage trend test (χ^2^ = 6.783, *p* = 0.009).

Analysis of the education levels showed that the illiterate group had the highest prevalence of TNs with 63.9%, followed by the primary school group with 56.8%, the secondary school group with 47.9%, high school/technical school/college group with 46.6%, the polytechnic school group with 37.4%, and the and undergraduate and above group with 32.2%. There was a significant difference in the prevalence of TNs among different education levels (χ^2^ = 106.035, *p* < 0.001). As the level of education increased, the prevalence of nodules decreased, as showed by the Cochran–Armitage trend test (χ^2^ = 96.799, *p* < 0.001) (Table 4).

By logistic regression, we found that age (*p* < 0.001), the female sex (*p* < 0.001), BMI (*p* = 0.006), and smoking (*p* = 0.017) were independently associated with the prevalence of TNs. Alcohol drinking, education level, taste preferences, seafood consumption habits, physical labor intensity, and physical exercise intensity had no independent correlation to the prevalence of TNs. The results showed that age (OR = 1.039 (1.034–1.044)), the female sex (OR = 1.789 (1.527–2.097)), BMI (OR = 1.019 (1.005–1.034)), and current smoking habits (OR = 1.246 (1.046–1.483)) were independent risk factors for TNs (Table 5).

## 4. Discussion

In recent years, the prevalence of TNs significantly increased. This survey was conducted on individuals over 18 years of age from seven communities in Beijing. It showed that the overall prevalence of TNs in the population was 49.0%. According to the literature, the prevalence was 67% in North America [10], 27% in Finland, 19% in Belgium, and 17% in Brazil [11]. The differences may be due to age, gender, inheritance, living environment, eating habits, and iodine nutritional status, among others [12,13,14].

This survey showed that the prevalence of TNs increased significantly with age, which is consistent with previous studies [15,16,17]. The prevalence among Japanese women over 40 years of age was 35.3%, and increased with age [18]. In another iodine-deficient area, solitary nodule prevalence remained stable independent of age, while the prevalence of multiple nodules increased with age [19,20]. The prevalence of TNs increased with age, which is likely to be due to degenerative changes occurring in the thyroid.

The prevalence of TNs was 52.5% in females, which was significantly higher, compared to the 42.7% for males. Logistic regression proved that the female sex was an independent risk factor for the development of TNs. Previous reports have also revealed that women were more likely to suffer from TNs than men [21,22]. Clinical studies have shown that the prevalence in pregnant and fertile women was much higher [23,24], suggesting that gender differences might be due to the combined effect of estrogen and progesterone. Since estrogen has an effect on the thyroid gland and stimulates thyrotropin (TSH) generation [25,26], and both normal and neoplastic thyroid tissues have estrogen receptor expression [27], it can be deduced that estrogen plays a possible role in the growth of thyroid cells and nodule formation. It was demonstrated in *in vitro* studies that 17β-estradiol may stimulate the growth of normal thyroid cells [28] and that thyroid follicular cells contained functional estrogen receptors [29]. Moreover, a previous study illustrated that estrogen could stimulate the growth of benign and malignant thyroid cells by activating the mitogen-activated protein kinase pathway [30]. However, Ceresini *et al.* [31] reported that the administration of estrogen for one year did not affect thyroid volume or the number and volume of thyroid nodules in postmenopausal women. Kim *et al.* [32] also reported that uterine fibroids were independently associated with the occurrence of TNs, although the study was more focused on premenopausal women. However, systemic estradiol levels had an inverse correlation with the incidence of TNs. This study hypothesized that, similar to uterine fibroids [33], the thyroid might have intracrine or paracrine estrogen responsiveness and *in-situ* estrogen synthesis [34,35]. The internal mechanisms of gender difference associated with TNs need to be further investigated.

In our study, a current smoking habit was demonstrated to be an independent lifestyle risk factor for thyroid nodules by logistic regression (OR = 1.246 (1.046–1.483)). Some previous studies have reported higher goiter prevalence and higher thyroid volumes among smokers compared to non-smokers [36,37]. Meanwhile, differing results were also published [38]. A discrepancy might be explained by differences in iodine status, as the association seems to be stronger in iodine-deficient areas [39]. Less research has focused on the association between TNs and smoking habits. We found a positive association of smoking with TNs in seven communities in Beijing, which is consistent with previous findings [40,41]. Several pathological mechanisms for the effect of smoking on thyroid morphology and function have been suggested. Thiocyanate, a degradation product of cyanide in tobacco smoke, is the major mediator of the goitrogenic effect of tobacco smoke. Thiocyanate acting as a competitive inhibitor inhibits iodine uptake and organification. It probably increases the prevalence of TNs and goiter by mimicking iodine deficiency. Smoking also stimulates thyroid hormone turnover, increases iodine demands and relative iodine deficiency, inhibits peripheral deiodinase activity, and has a direct effect on the pituitary. However, a Denmark study [42] reported that smoking was associated with an increased prevalence of thyroid multinodularity (OR = 1.9 (1.4–2.5)), but not with an increased prevalence of solitary thyroid nodules. The association with multinodularity was also not statistically significant following salt iodization [43]. Karatoprak *et al.* [44] observed that smoking had no effect on TN formation in iodine-sufficient regions like Istanbul. Furthermore, well-designed studies taking the amount and duration of smoking into account should be conducted to fully elucidate this relationship. Interestingly, we found no difference in the occurrence of TNs between drinkers and non-drinkers. A cross-sectional population study [45] found that a lower prevalence of a solitary nodule was associated with increased levels of alcohol consumption. Confirmatory studies are required.

The logistic regression analysis showed that BMI was another independent risk factor for TNs. A study from Korea [4] also reported that a higher BMI was associated with a higher frequency of TNs in women. Another study [5] showed that a greater prevalence of nodules in obese male subjects. The precise mechanisms were not entirely clear. The main hypothesis is that the association between obesity and TNs is by means of insulin resistance (IR). In a previous report by Rezzonico *et al.* [46], patients with IR have a higher risk of formation of TNs. Higher circulating levels of insulin might cause increased thyroid proliferation. Another hypothesis is some humoral or hormonal mediators like leptin from adipose tissue stimulate the hypothalamus-pituitary-thyroid axis [6]. Further studies are necessary to illuminate the association between obesity and TNs.

Our results showed that an increased frequency of seafood consumption was associated with a decreased prevalence of TNs. However, the logistic regression analysis did not show an independent association between the frequency of seafood intake and prevalence of TNs. A similar trend was evident in a study conducted in Belgium [47], which reported that TN prevalence in Wallonia was higher than that in Flanders, while the consumption of seafood was significantly lower in Wallonia than in Flanders. The author believed that the longer distance of Wallonia to the sea than Flanders resulted in the lower iodine content of the local food. The difference in iodine content was the most plausible explanation for the difference in thyroid nodular prevalence. Our regression analysis proved that the frequency of seafood consumption was not an independent factor in the development of TNs. However, it may be affected by iodine intake, living habits, and other confounding factors. Further evaluation of the iodine nutritional status of the participants could help clarify the argument. Concerning the relationship between iodine nutritional status and TNs, Szabolcs *et al.* [48] showed that in three elderly Hungarian groups with deficient, adequate, and excessive iodine intake levels who underwent ultrasound TN detection, the prevalence rates were 20.2%, 16.2%, and 3.3%, respectively. Prevalence rates increased with a decreased iodine intake. On the contrary, Hu *et al.* [49] reported that the prevalence of TNs in deficient, adequate, and excessive iodine areas were 12.6%, 10.2%, and 10.8%, respectively, with no significant difference. Follow-up studies need to be conducted in order to fully reveal the relationship between iodine intake and the prevalence of TNs.

Univariate analysis showed that, with decreasing physical labor and physical exercise intensities, the prevalence of TNs increased. A significant negative correlation was observed in both instances as shown by the trend test. Leng *et al.* [50] reported that the prevalence of TNs in men who did not exercise or exercised rarely was significantly higher. In women, no such correlation was observed. We showed by multi-factor correction that labor and exercise were not independent factors in the development of TNs. However, these results indicate that other metabolic factors associated with labor and exercise may be relevant to the development of TNs.

Furthermore, we showed that the prevalence of TNs decreased with as education level increased; however, regression analysis showed this was not an independent factor. This may in part be attributed to the younger population often having higher education levels, the difference in smoking habits, and the methods of using iodinated salt. Knudsen *et al.* [51] also reported that the higher the education level, the smaller the thyroid volume, and the lower the prevalence of multiple nodules. On the other hand, the associations diminished markedly if an adjustment was made for smoking habits, alcohol consumption, and iodine intake.

Our study includes a large sample size for the prevalence of TNs in North China, and we have conducted a comprehensive analysis on the relationship between the prevalence of TNs and multiple lifestyle factors. The present study also has several limitations. First, there was no dose-response analysis between the amount and timing of both smoking and TN prevalence. Second, we did not assess the iodine nutritional status. A follow-up study that takes these factors into account is needed in the future.

## 5. Conclusions

In summary, our study determined the prevalence of TNs in Beijing, their epidemiological characteristics, and their association to lifestyle factors. The results showed that TN prevalence in Beijing was high. It was higher in females than in males, and increased with older age. There were more multiple nodules than solitary nodules. Age and the female sex were independently associated with the prevalence of TNs. Smoking and BMI were the independent lifestyle risk factors. These findings have provided some insights for clinical practice that can aid in the improvement of preventive strategies for thyroid nodules.

## Figures and Tables

**Table 1:** General characteristic of subjects.

Parameters	Total	Male	Female	*p*
*n* (%)	6324 (100)	2264 (35.8)	4060 (64.2)	
Age (years)	52.15 ± 11.58	52.88 ± 11.38	51.75 ± 11.68	<0.001
History of coronary heart disease (%)	7.8	6.0	8.8	<0.001
History of diabetes (%)	11.5	11.9	11.3	0.511
History of hypertension (%)	30.2	30.8	29.8	0.411
History of dyslipidemia (%)	10.5	10.5	10.5	0.962
Smoker (%)	30.0	72.6	6.2	<0.001
Drinker (%)	27.2	67.9	4.4	<0.001
BMI (kg/m^2^)	27.02 ± 3.98	27.18 ± 3.78	26.93 ± 4.08	0.014

*p*: Male compared to female.

**Table 2:** The prevalence of thyroid nodules in subjects.

Prevalence	Male		Female		Total	
	*n*	%	*n*	%	*n*	%
Solitary nodules	395	40.8	704	33.0	1099	35.5
Multiple nodules	572	59.2	1429	67.0	2001	64.5
Total	967	42.7	2133	52.5	3100	49.0

**Table 3:** Relationship of smoking and drinking to the prevalence of thyroid nodules stratified by gender.

Parameters		Male		*p*	Female		*p*
		TN (+) (%)	TN (−) (%)		TN (+) (%)	TN (−) (%)	
Smoking History	Current smokers	43.7	56.3	0.376	61.4	38.6	0.007
	Former smokers	42.1	57.9		64.4	35.6	
	Non-smokers	40.3	59.7		51.9	48.1	
	Missing	3.9					
Drinking History	Current drinkers	43.1	56.9	0.376	49.0	51.0	0.061
	Former drinkers	45.4	54.6		75.0	25.0	
	Non-drinkers	40.5	59.5		52.5	47.5	
	Missing	4.1					

*p*: TN (+) group compared to TN (−) group.

**Table 4:** Comparison of the prevalence rates of thyroid nodules in different lifestyle subgroups.

Parameters	Categories	TN (+) (%)	TN (−) (%)	*p*
Taste preference	Salty	48.6	51.4	0.854
	Average	49.4	50.6	
	Less Salty	48.8	51.2	
	Missing	4.1		
Seafood intake	Frequently	46.1	53.9	0.048
	Occasionally	48.5	51.5	
	Never	52.4	47.6	
	Missing	4.1		
Physical labor intensity	Very light	50.5	49.5	0.001
	Light	45.4	54.6	
	Moderate	43.1	56.9	
	Heavy	25.0	75.0	
	Missing	4.4		
Physical exercise intensity	Mild	49.6	50.4	0.01
	Moderate	47.5	52.5	
	High	34.4	65.6	
	Missing	8.3		
Education level	Illiteracy	63.9	36.1	<0.001
	Primary school	56.8	43.2	
	Secondary school	47.9	52.1	
	High school	46.6	53.4	
	Polytechnic school	37.4	62.6	
	Undergraduate and above	32.2	67.8	
	Missing	1.2		

*p*: TN (+) group compared to TN (−) group.

**Table 5:** Logistic regression analysis with possible risk factors as independent variables and the prevalence of thyroid nodules as a dependent variable.

Variables	β	SE	*p*	OR	95% CI
Age	0.038	0.003	<0.001	1.039	1.034–1.044
Female	0.582	0.081	<0.001	1.789	1.527–2.097
BMI	0.019	0.007	0.006	1.019	1.005–1.034
Smoking			0.017		
Former Smokers	−0.045	0.129	0.729	0.956	0.743–1.231
Current Smokers	0.220	0.089	0.014	1.246	1.046–1.483

β: logistic regression coefficient; SE: standard error; OR: odds ratio; CI: confidence interval.

## Abbreviations

The following abbreviations are used in this manuscript:

PLA	People’s Liberation Army
TN	Thyroid nodules
WHO	World Health Organization
OR	Odds ratio
BMI	Body mass index
TSH	Thyrotropin
IR	Insulin resistance
